# High mortality rates after radical cystectomy: we must have acceptable protocols and consider the rationale of cutaneous ureterostomy for high-risk patients

**DOI:** 10.1590/S1677-5538.IBJU.2019.06.03

**Published:** 2019-12-17

**Affiliations:** 1 Disciplina de Urologia, Faculdade de Medicina do ABC, Santo André, SP, Brasil; 2 Fundacion Puigvert Universitat Autonoma de Barcelona, Espanha

Bladder cancer is a common disease, and for T2-T4 stages, radical cystectomy is the first treatment option (1). An interesting Swedish study has evaluated the natural history of urothelial bladder cancer. After 6 months of diagnosis, 38% of patients develop metastasis if untreated (2). Five-year Cancer-specific survival is as low as 14% in such scenery, and overall survival is only 5% (2). On the other hand, if treated these patients have a 5-year CSS and OS of 60% and 48% respectively (2). Radical cystectomy is, therefore, the first option, as it is also stated by the EAU, NCCN, AUA /ASCO / ASTRO / SUO guidelines/consensus (3-5) is associated with a significant survival gain in comparison to observation (2), to multiple resections, chemotherapy or radiotherapy (6). In patients with stage II disease, cystectomy is associated with a three-fold increase in survival, increasing mean overall survival from 16 to 45 months (6). In a SEER study evaluating 328,560 patients, radical cystectomy and chemotherapy were the only factors associated with improvements in survival (7). Trimodal “bladder-sparing” approaches that combine maximal transurethral resection, chemotherapy, and radiotherapy or neoadjuvant chemotherapy with partial cystectomy are an option but only for a small percentage of patients (3).

However, if we analyze data carefully, the guideline recommendations are rarely followed. In a SEER study that evaluated 6.737 patients in the USA with stage II disease (non-metastatic, muscle-invasive bladder cancer), only 8.3% underwent radical cystectomy (8). The odds of an octogenarian to undergo radical cystectomy in the USA is five-times lower than a young patient (8). Hispanic origin, Afro-American origin, and lower scholar level patients are also less treated properly when they have muscle-invasive bladder cancer (8). According to a very interesting study that evaluated 27,578 patients from the SEER, only 6% of patients with muscle-invasive bladder cancer (pT2-pT4) in the USA underwent radical cystectomy between 2007 and 2013 (8). Less than 19% of patients with pT2 disease in the USA undergo radical cystectomy (9).

And why does this happen? The answer is because radical cystectomy is associated with high morbidity and mortality rates. When described in the late 1940s, radical cystectomy was associated with a perioperative mortality of 33% (10). In the 1970s perioperative mortality decreased to 11% (and remained around 2.1% to 4.7% after the 1980s) (11). Analyzing mortality after radical cystectomy is a slippery slope, as demonstrated in Table-1. Studies report distinctive data. In-hospital mortality is lower than 30-day mortality, which is two to three-fold lower than 90-day mortality. And these numbers vary widely (1, 12, 13).

**Table 1:** Mortality after radical cystectomy in distinctive settings.

Author	N	IHM	30-day	90-day	Setting
Barbieri, 2019, (14)	6,728	0.54%	–	–	Large volume academic centers
Udovicich, 2017. (15)	803	2.2%	–	–	Victoria state, Australia
Afshar, 2018, (12)	15,292		2.7%	7%	English database
Afshar, 2018, (19)			1.5%	4%	English database (after centralization program)
Waingankar, 2019, (1)	47,028		3.0%	8.2%	SEER, USA
Dell'Oglio, 2019, (16)	7,076	–	–	10.7%	SEER, USA
Timoteo, 2019, (18)	5.097	7.38%	–	–	DATASUS, Brasil
DATASUS, 2019	1,377	8.5%	–	–	DATASUS, state of SP
DATASUS, 2019	161	8.1%	–		DATASUS, institution 1, São Paulo
DATASUS, 2019	84	6.0%	–		DATASUS, institution 2, São Paulo
DATASUS, 2019	71	11.3%	–		DATASUS, institution 3, São Paulo
DATASUS, 2019	53	15.9%	–		DATASUS, institution 4, São Paulo
DATASUS, 2019	81	14.8%	–	–	DATASUS, suburban institutions of São Paulo

**IHM** = in-hospital mortality

In large volume academic referral centers in the USA, in-hospital mortality after 6,728 cystectomies was as low as 0.54% (14). This results are impressive, and can be either effect of experience but also to a high selection bias. If we go to other less selected settings, things start to change. An Australian epidemiologic study has observed a 2.2% rate after 803 surgeries (15). A British study evaluated 15,292 patients and have observed a mortality rate of 2.7% after 30 days and of 7% after 90 months (12). In a large epidemiologic study evaluating SEER data, the mortality rate of 47,028 patients who underwent radical cystectomy in 1,162 centers was 3% after 30 days and 8.2% after 90 days (1). In another large SEER study with 7,076 patients, 90-day mortality ranged from 10.75% to 13.1% (16). In Spain a national database study that has evaluated 7,999 patients found a 4.7% in-hospital mortality and 6.2% 90-day mortality rate (17).

In developing countries, reality seems to be much tougher in the public scenario. We have recently published a study demonstrating a 7.38% in-hospital mortality rate, with a wide variation according to geographic regions, varying from 6.2% in the south to 28.6% in certain regions of the North of Brazil (18). Data from the public health system (DATASUS) in the State of São Paulo, the most populated and wealthier state of Brazil, between 2008-2018 demonstrated amid 1,377 radical cystectomies reported, and 117 in-hospital deaths (8.5%). In the five largest academic institutions in the city of São Paulo that treat exclusively patients from the public health system, in-hospital mortality varied from 6.0% to 15.9% (18). And it is important to state that these numbers represent in-hospital mortality. Probably 90-day mortality is even higher.

## Ureterostomy as a strategy to reduce mortality?

Even though the AUA / ASCO / ASTRO /SUO guidelines recommend as first-line treatment for MIBC not only radical cystectomy but also urinary diversion using intestinal segments (ileal conduit, continent cutaneous diversion or orthotopic neobladder) as standard treatments (4), this is not what is currently done in more than 90% of patients with MIBC in the USA, where only a fraction of patients with MIBC undergo radical cystectomy. Data evaluating a series of patients undergoing radical cystectomy in developing countries are scarce in the literature, mainly because mortality rates are unacceptable in most public large volume centers. Some key points that have been adopted by European centers might urgently be adopted in the developing world (3). They include centralization of treatment and surgeries to referral centers (19, 20), proper patient preparation and adequate choice of diversion for each patient (12, 21). In high risk patients, cutaneous ureterostomy might be also considered as a good alternative (3, 22). Several authors have demonstrated a significant reduction in complications and mortality with cutaneous ureterostomy after radical cystectomy mainly in patients with high risk for complications; it reduces the duration of the surgery and all the complications related to bowel manipulation, thus decreasing the risk of mortality in patients with comorbidities (22-25).

Some key-points are that not every patient with MIBC is a candidate for radical cystectomy, but also not every patient suitable for radical cystectomy is a candidate for urinary diversion with intestinal segments. Urinary diversion using intestinal segments is responsible for a large number of these deaths (23). Even though this insight has been widely published before (22-25), mortality rates at many Centers are still high. If in one hand we discuss robotic use (21, 26-28) that is been used in the vast majority of patients treated with muscle-invasive bladder cancer, we have to talk about this problem in our journals, meetings, boards, and discussions and bring better solutions to be sure that we are first of all not harming our patients. We have to properly perform a correct pre/intra/postop management of the patients and therapeutic decisions might be in accordance to comorbidities and life expectancy.
